# Vanillic Acid Inhibited the Induced Glycation Using In Vitro and In Vivo Models

**DOI:** 10.1155/2022/7119256

**Published:** 2022-11-18

**Authors:** Amani Alhadid, Yasser Bustanji, Amani Harb, Yusuf Al-Hiari, Shtaywy Abdalla

**Affiliations:** ^1^Department of Pharmacy, Faculty of Pharmacy, Al-Zaytoonah University of Jordan, Amman, Jordan; ^2^Department of Biopharmaceuticals and Clinical Pharmacy, School of Pharmacy, The University of Jordan, Amman 11942, Jordan; ^3^Department of Basic Medical Sciences, College of Medicine, University of Sharjeh, Sharjeh 27272, UAE; ^4^Department of Basic Sciences, Faculty of Arts and Sciences, Al-Ahliyya Amman University, Amman, Jordan; ^5^Department of Pharmaceutical Sciences, School of Pharmacy, The University of Jordan, Amman 11942, Jordan; ^6^Department of Biological Sciences, School of Science, The University of Jordan, Amman 11942, Jordan

## Abstract

**Background:**

Glycation is implicated in the pathophysiology of many diseases, including diabetes, cancer, neurodegenerative diseases, and aging. Several natural and synthetic compounds were investigated for their antiglycation activity. We evaluated the antiglycation effect of vanillic acid (VA) using in vitro and in vivo experimental models.

**Methods:**

In vitro, bovine serum albumin (BSA) (50 mg/ml) was incubated with glucose (50 mM) with or without VA at 1.0–100 mM for 1 week at 37°C, and then, excitation/emission fluorescence was measured at 370/440 nm to determine glycation inhibition. The cytoprotective effect of VA was evaluated using RAW 264.7 cells incubated with or without VA at 7.8–500 *μ*M along with 100–400 *μ*M of methylglyoxal for 48 hours, and cell viability was determined using the MTT assay. Aminoguanidine (AMG) was used as a positive control in both in vitro and cell culture experiments. In vivo, 52 streptozotocin-induced diabetic rats were randomly assigned to 4 groups and treated with 0, 1.5, 4.5, or 15 mg/kg VA for four weeks. Serum fructosamine and blood glycosylated hemoglobin (HbA1c) were then measured, and advanced glycation end-products (AGEs) were detected in the kidneys and the skin of deboned tails using an immunohistochemistry assay.

**Results:**

VA caused a concentration-dependent effect against BSA glycation (IC50 of 45.53 mM vs. 5.09 mM for AMG). VA enhanced cell viability at all concentrations of VA and methylglyoxal. VA did not affect serum fructosamine or blood HbA1c levels, although it markedly decreased AGEs in the kidney in a dose-dependent manner and decreased AGEs in the skin of deboned tail tissues.

**Conclusion:**

VA had significant antiglycation activity at cellular and long-term glycation.

## 1. Introduction

Glycation is considered one of the most detrimental processes taking place in the body. Chemically, glycation is a multistep process where reducing sugars react nonenzymatically with amine groups of amino acids and other macromolecules such as nucleic acids and lipids, altering the structure and function of these biological compounds, inducing pathological events, and producing a plethora of nonbiological compounds with damaging adverse effects [1].

Glycation leads to serious pathological effects, including alteration of protein structure and function, inflammation, oxidative stress, carbonyl stress, cytotoxicity, and genotoxicity. In vivo glycation has been found to be implicated in the pathophysiology of many diseases, including diabetes, cancer, vascular diseases, neurodegenerative diseases, inflammatory diseases, and aging [2, 3].

The first phase of glycation includes the formation of a Schiff base due to interaction of sugars with the amino groups of proteins, followed by rearrangement, leading to Amadori products. The second phase includes the formation of carbonyl intermediates like glyoxal, methylglyoxal, and 3-deoxyglucosone, and the third phase includes the conversion of the products into advanced glycation end-products (AGEs), where sugars may cross-link components in one or different protein molecules, thereby modifying their function [4, 5].

In addition to their impact on proteins and extracellular matrix, AGEs can interact with receptors like RAGE, a multiligand receptor, to trigger various intracellular signaling cascades, stimulating JAK/Stat, P38 MAPK, ERK, and JNK, leading to the activation of NF*κ*B, thus causing increased expression of cytokines, growth factors, and adhesion molecules [6]. In addition, RAGE engagement has been reported to result in the progression of different types of cancer, like pancreatic cancer [7].

Being the main mechanism that contributes to the pathogenesis of many maladies, the clinical importance of combating glycation is greatly emphasized. Substantial research has been directed towards exploring compounds with antiglycation potential in an attempt to prevent or attenuate the extent of glycation and, subsequently, its degenerative effects. So far, a number of compounds with appreciable antiglycation activity have been identified; however, none has been approved for clinical use either because of insufficient efficacy or safety concerns [8–10].

Many natural compounds, especially polyphenols, have been found to efficiently inhibit protein glycation in vitro. Their action in vivo is more problematic due to various reasons, including limited bioavailability, although some progress has been reported using green nanoparticles [11]. Nevertheless, several positive effects of natural antioxidants against glycation or its consequences have been reported [8–13]. While the mechanisms of their action may go beyond direct inhibition of glycation, there are reasons to expect that natural compounds may prevent the adverse effects of protein glycation and, in consequence, ameliorate certain degenerative diseases and delay aging [9]. Vlassopoulos and Combet reviewed the antiglycation effect of polyphenols and concluded that protein pretreatment with phenolic acids is an important regulator of subsequent glycation in physiological systems [14].

In this study, we have investigated the antiglycation activity of vanillic acid, a phenolic acid with many biological activities. Vanillic acid (4-hydroxy-3-methoxybenzoic acid; VA), a derivative of several edible plants and fruits and used in traditional Chinese medicine, has been reported to have antioxidant, antimicrobial, analgesic, and anti-inflammatory effects [15, 16]. The antioxidative activity of VA involves free radical scavenging activity, reducing power, and inhibition of lipid peroxidation. Additionally, VA has been found to reduce lipid peroxidation and restore enzymatic and nonenzymatic antioxidants in the plasma of hypertensive rats [17].

In view of these bioactive properties, we aimed to investigate the antiglycation activity of VA by examining its ability to suppress induced glycation using in vitro and in vivo experimental models.

## 2. Materials and Methods

### 2.1. In Vitro Phase

This assay was done as previously described by Rahbar et al. with minimal modifications [18]. BSA, sodium azide, and glucose were dissolved in Na phosphate buffer (pH 7.4; 0.2 M), to a final concentration of 50 mg/ml BSA, 0.02% sodium azide, and 50 mM glucose monohydrate. All solutions were filter sterilized, and the experiments were done under aseptic conditions to prevent microbial contamination. Reaction mixtures were prepared by adding BSA, sodium azide, and glucose solutions with or without VA (1.0, 1.6, 3.1, 6.2, 12.5, 25, 50, and 100 mM). The positive control aminoguanidine (AMG) was also incubated with the reaction mixture at the concentrations 1.0, 1.6, 3.1, 6.2, 12.5, 25, 50, and 100 mM. The reaction mixtures were incubated in 24 well sterile plates (1 ml/well) at 37°C for 7 days. On day 7, samples of 200 *μ*L of the reaction mixture were aspirated into 96-well black plates, and their fluorescence was examined at 370/440 nm excitation/emission wavelengths using a fluorescence spectrophotometer (FLx800, BioTek). The activity of VA was assessed by calculating the percentage of inhibition versus the negative control (reaction mixture without VA) according to the formula [18]

(1) $$\begin{array}{c} \left\{1-\left(\frac{fluorescence\;of\;sample\;compound}{fluorescence\;of\;negative\;control}\right)\right\}\times 100\%\text{.} \end{array}$$

The half maximal inhibitory concentration (IC50) for VA was then found using GraphPad Prism, version 5.01. In order to exclude the effect of any inherent fluorescence of VA, blank samples of VA dissolved in the solvent only at the designated 8 concentrations were examined at the same excitation/emission wavelengths (370/440 nm) used in the assay. The assay was done in duplicate, with 3 readings for each sample in each run.

### 2.2. Cytoprotective Effects of VA

A continuous cell line of murine macrophages (RAW 264.7) was cultured in DMEM media enriched with 10% fetal bovine serum (FBS), streptomycin sulfate (100 mg/ml), penicillin (100 U/ml), gentamicin (50 *μ*g/ml), beta-mercaptoethanol (50 *μ*M), HEPES buffer (10 mM), and L-glutamine (2 mM). The cells were cultured routinely, harvested biweekly using 1% trypsin-EDTA, seeded at 10^4^ cells/well in 96-well tissue culture plates, and incubated at 37°C under 5% CO_2_ overnight to allow for adhesion. After 12 hours of incubation, the cells were treated with VA (0, 7.8, 15.6, 31.25, 62.5, 125, 250, and 500 *μ*M) with the glycating agent, the *α*-dicarbonyl methylglyoxal (MGO; 0, 100, 200, 300, 400 *μ*M). VA was added 20 minutes prior to MGO. VA was dissolved in 3% ethanol, such that the maximum final concentration of ethanol on cells did not exceed 1%. The following control tests were conducted: (1) cells incubated with the media only and the maximum concentration of ethanol used (vehicle-treated cells); (2) cells incubated with MGO without VA (negative control); (3) cells incubated with 1 mM AMG without VA (positive control) [19]. In another set of experiments, cells were incubated with VA only at the used concentrations (0–500 *μ*M of VA) to assess the cytotoxicity of VA.

Cells were then incubated for 48 h and assessed for viability using the MTT test [20]. The absorbance was read at 570 nm and 630 nm as reference wavelengths using an absorbance plate reader (BioTek, USA). The percentage of treated viable cells was calculated in reference to vehicle-treated cells (0 MGO, 0 VA). The activity of VA was assessed by calculating the percentage of viable cells according to the following equation:

(2) $$\begin{array}{c} VA\;activity=\left\{1-\left(\frac{A1-A2}{A1}\right)\right\}\times 100\%\text{,} \end{array}$$

where *A*1 is the mean absorbance of vehicle-treated cells (0 VA, 0 MGO), and *A*2 is the mean absorbance of treated cells (VA and MGO).

Cell viability of percentages less than 70% was considered the toxicity cutoff point [21]. The assay was done in duplicate, with 3 readings for each sample in each run.

### 2.3. In Vivo Phase

Seventy-four Sprague Dawley male rats weighing 250–300 g were individually caged, labeled, and kept in the animal house for two weeks for acclimatization and fed regular chow diet (Hammoudeh Farms, Jordan) ad libitum except when they were fasted prior to streptozotocin (STZ) injection. Animals were handled according to the regulations of the Deanship of Academic Research at the University of Jordan. The experimental protocols were also approved by the Research Committee of the School of Science at the University.

#### 2.3.1. Induction of Diabetes

Rats were fasted for 16 h and then injected intraperitoneally (i.p.) with freshly prepared STZ (35 mg/kg dissolved in 0.1 M cold citrate buffer, pH 4.5) to induce partial diabetes. Other 7 rats were given the vehicle (citrate buffer) and served as a normal control group (group 1). Forty-eight h later, tail blood sugar was checked using a commercial glucometer (Roche, Germany), and rats with random blood sugar above 200 mg/dl were considered diabetic and were included in the experimental design for subsequent treatments [22].

#### 2.3.2. VA Treatment

Diabetic rats were randomly allocated into 4 groups (13 animals each): one diabetic control group was injected with distilled water (group 2), and 3 treatment groups were injected i.p. with VA at doses of 1.5 mg/kg, 4.5 mg/kg, or 15 mg/kg (groups 3–5, respectively) for 4 weeks on a daily basis. Treatment with VA was initiated 48 h after STZ injection (day 1) and continued till day 28. Blood sugar was measured weekly to ensure diabetes continuation, and body weight was monitored weekly to adjust the VA dose accordingly.

On day 28 of treatment, rats were sacrificed, and blood samples were collected in EDTA tubes for subsequent HbA1c analysis and in plain tubes for subsequent serum fructosamine analysis. Longitudinal and cross-sections of the right kidney and the skin of deboned tails, respectively, were made and preserved in 10% formalin in normal saline fixative for the immunohistochemistry (IHC) assay.

#### 2.3.3. Determination of Fructosamine (FA)

The assay was performed using an FA assay kit (Bioscientific, TX, USA) following the manufacturer's instructions. Briefly, 15 *μ*L of serum or standard was added in duplicates to prewarmed 300 *μ*L of fructosamine reagent using 96-well plates, and absorbance was instantly measured at 550 nm (A1). The plate was then incubated at 37°C for 15 min, and absorbance was remeasured at the same wavelength (A2). The change in absorbance (A2-A1) was plotted against the concentration of different dilutions of the standard solution, and the concentration of FA in the samples was accordingly determined.

#### 2.3.4. Determination of Glycosylated Hemoglobin (HbA1c)

The determination of glycosylated hemoglobin HbA1c was performed using the turbidimetric inhibition immunoassay on haemolysed whole blood samples using the Tina-quant A1c kit (Roche, Switzerland). The assay was done according to the manufacturer's instructions. The assay principle is based on the reaction of glycohemoglobin (HbA1c) in the sample with anti-HbA1c antibodies to form antigen-antibody complexes that can be determined turbidimetrically. Liberated hemoglobin from the haemolysed blood in the sample is converted to a derivative that has a characteristic absorption spectrum measured bichromatically. The final result is expressed as % HbA1c.

#### 2.3.5. AGEs Detection in the Kidney and the Skin of Deboned Tail Tissues

Detection of AGEs in the tissues of the kidney and the skin of deboned tails was performed using an immunohistochemical assay (IHC). Longitudinal sections of the right kidney and cross sections of the tail tips of rats were collected at the end of the treatment period (day 28). The specimens were preserved in 10% formalin in normal saline fixative for a subsequent IHC assay. Specimens were put in cassettes, dehydrated, cleared, and infiltrated with wax using an automated tissue processor. The cassettes were then embedded in paraffin and cut into 4 *μ*m thick slices by microtome (Lipshaw, USA). Formalin-fixedparaffin-embedded cuts were then mounted onto adhesive slides, heated at 70°C for 1 hr., dewaxed, rehydrated, incubated with 3% hydrogen peroxide for 25 min, and washed with PBS. The sections were then pretreated with Tris-EDTA retrieval solution (pH 9) at 95°C for 15 min, washed with PBS, blocked with a protein blocking agent for 5 min, and subsequently incubated with anti-AGE rabbit polyclonal antibody (ab23722, Abcam, UK) at a dilution of 1/50 for 1 hr at room temperature. The antibody-antigen interaction was then assayed using the EXPOSE rabbit-specific HRP/DAB detection IHC kit (Abcam, UK). The sections were then incubated with goat anti-rabbit HRP conjugate for 15 min at room temperature, followed by immersion in the chromogen 3, 3′-diaminobenzidine tetrahydrochloride (DAB). Finally, sections were counterstained with 50% hematoxylin and mounted with distrene, plasticizer, and xylene (DPX).

Immuno-stained sections were blindly examined by a histopathologist at the University Hospital. Having no pre-established IHC scoring system for AGEs, a self-adopted scoring system for grading the extent of glycation as indicated by the stained spots of AGEs was developed by the histopathologist. The developed scoring system consisted of four grades: strongly positive (++), positive (+), slightly positive (+/−), and negative (−). Representative slides from each animal group were examined, and the extent of glycation was assessed relative to that of the diabetic control, which was considered a reference point with a strongly positive score.

Sections of arteriosclerotic plaques obtained from tissue cassettes for a 72-year-old patient stored in the histopathology lab of the university hospital were used with each run as a positive control, as indicated by the manufacturer of the antibody kit (ab23722, Abcam, UK). This positive control showed very strong positive reaction with diffused pattern in all performed runs. The slides were examined under a light microscope with a digital camera (Leica, Germany) using the software LASEZ 1.8.0, and representative pictures at different magnifications were captured.

### 2.4. Statistical Analysis

Data were presented as means ± SEM and were analyzed using one-way analysis of variance (ANOVA) followed by Dunnett's post-hoc test when comparison was made relative to a control. The values of IC50 for VA in the in vitro test were calculated assuming a nonlinear regression response. A student's *t*-test with Welch's correction was used to compare the effects of AMG and MGO. Differences were considered significant at *p* < 0.05. Data were analyzed using the statistical software GraphPad Prism (5.01).

## 3. Results

### 3.1. VA Inhibited Glucose-Induced BSA Glycation

**Figure 1:**
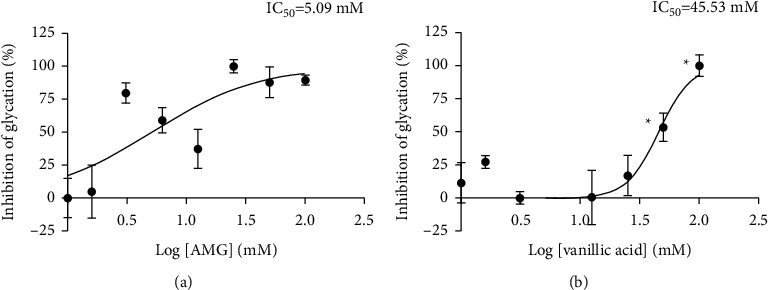
Concentration-response curves of glycation inhibition by AMG (a) and vanillic acid (b). The data represent the percentage of glycation inhibition relative to the control (0 mM AMG or 0 mM VA) and are expressed as mean ± SEM of duplicate tests with 3 readings for each sample. Data points with asterisks in B represent significant differences from the control (0 mM VA) (*p* < 0.05).

Figure 1 shows the effect of VA and the positive control AMG on glucose-induced BSA glycation. AMG caused remarkable inhibition of BSA glycation in all tested concentrations (*p* < 0.001). VA also significantly reduced BSA glycation at 50 and 100 mM (*p* < 0.001). Both VA and AMG showed a dose-dependent inhibition of glycation. The IC50 values for VA and AMG were 45.53 mM and 5.09 mM, respectively (*p* < 0.05).

### 3.2. VA Protected against MGO-Induced Cytotoxicity on RAW 264.7 Cells

**Figure 2:**
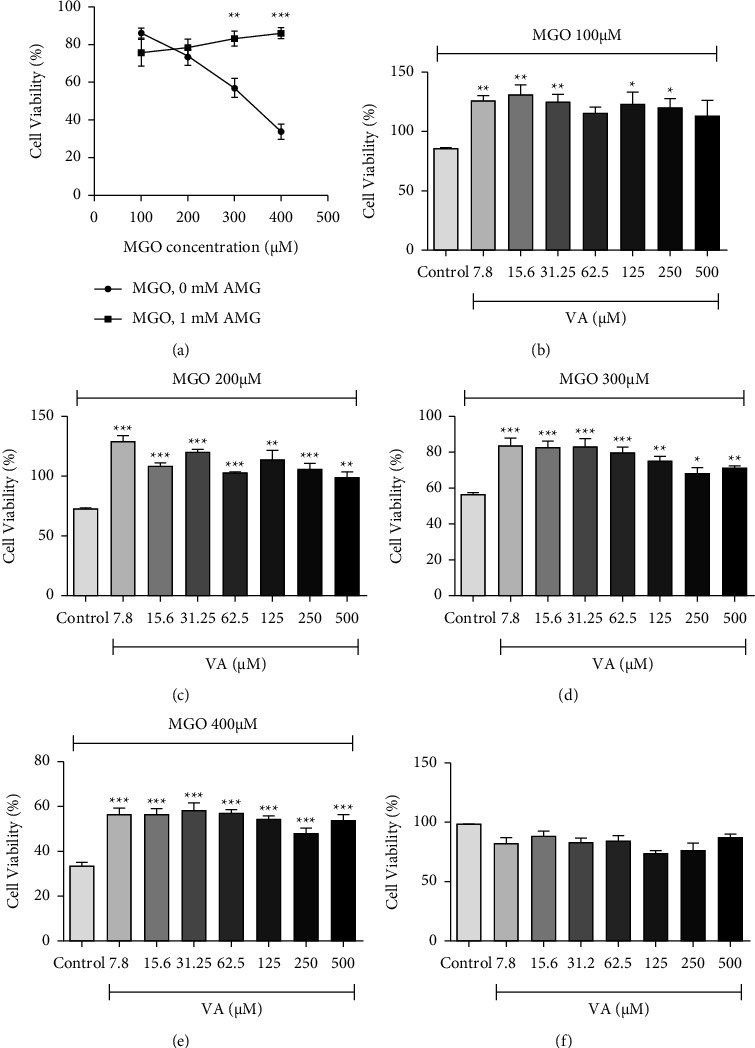
Effect of AMG and vanillic acid (VA) on viability of cells treated with MGO. (a) Viability of MGO-treated cells in the presence or absence of 1 mM AMG. Data points with asterisks indicate significant difference from the control (MGO-treated cells), ^*∗∗*^*p* < 0.01, ^*∗∗∗*^*p* < 0.001. (b)–(e) Effect of VA (7.8–500 *μ*M) on viability of cells treated with the indicated concentrations of MGO, expressed as percentages from the vehicle-treated cells. Columns with asterisks indicate significant difference from the control (MGO-treated, 0 *μ*M VA), ^*∗*^*p* <  0.05, ^*∗∗*^*p* < 0.01, ^*∗∗∗*^*p* < 0.001. (f) Viability of cells treated with VA (7.8–500 *μ*M) expressed as percentages from control (vehicle-treated cells, 0 *μ*M VA). All data are presented as mean ± SEM for two independent runs and three readings for each sample.

Figure 2 shows the effect of 1 mM AMG and different concentrations of VA on RAW 264.7 cells that have been treated with different concentrations (100, 200, 300, and 400 *μ*M) of the glycating agent MGO. MGO inhibited cell viability in a concentration-dependent manner, whereas AMG reversed this inhibition (Figure 2(a)). VA, in concentrations ranging from 7.8 *μ*m to 500 *μ*M, significantly antagonized the inhibitory effect of MGO on cell viability (Figures 2(b)–2(e)). This effect of VA was more observable at the higher concentrations of MGO (Figures 2(c)–2(e)). VA caused significant enhancement in cell viability when compared to the control (0 *μ*M VA), in all concentrations of MGO used (*p* < 0.05). VA itself had no effect on RAW 264.7 cells' viability in any of the concentrations used (Figure 2(f)), as cell viability was >70% at all VA concentrations used.

### 3.3. VA Had No Effect on Fructosamine (FA) and HbA1c Levels

**Figure 3:**
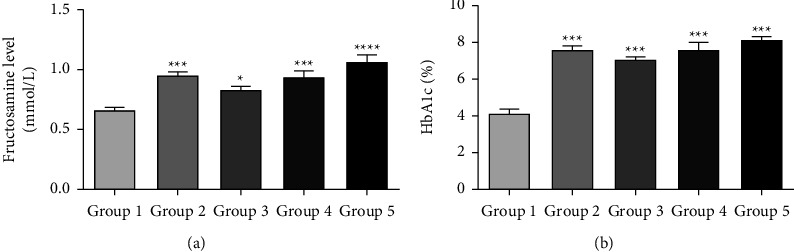
Fructosamine (a) and HbA1c (b) levels in the rat groups after treatment with VA for 4 weeks. Group 1 (nondiabetic, vehicle-treated, *n* = 7), group 2 (diabetic, vehicle-treated, *n* = 9), group 3 (1.5 mg/kg VA, *n* = 9), group 4 (4.5 mg/kg VA, *n* = 9), and group 5 (15 mg/kg VA, *n* = 7). Columns with asterisks indicate significant difference from the control group (group 1), ^*∗*^*p* <  0.05, ^*∗∗∗*^*p* < 0.001. Data are presented as mean ± SEM.

Figure 3 shows the effect of VA on the levels of fructosamine (FA) and glycosylated hemoglobin (HbA1c) in rats. FA level was significantly lower in the control rats (group 1, nondiabetic, vehicle-treated rats) compared to all diabetic rats (groups 2–5) (*p* < 0.05). No significant differences between any of the treatment groups (groups 3, 4, and 5) and the diabetic, nontreated group (group 2) could be found (*p* > 0.05).

HbA1c level was significantly lower in the control rats (group 1, nondiabetic, vehicle-treated rats) compared to all diabetic rats (groups 3, 4, and 5) (*p* < 0.001). No significant differences between any of the treatment groups (groups 3, 4, and 5) and the diabetic, nontreated group (group 2) could be found (*p* > 0.05).

### 3.4. VA Reduced AGEs Formation in the Immunohistochemical Assay of the Kidneys

**Figure 4:**
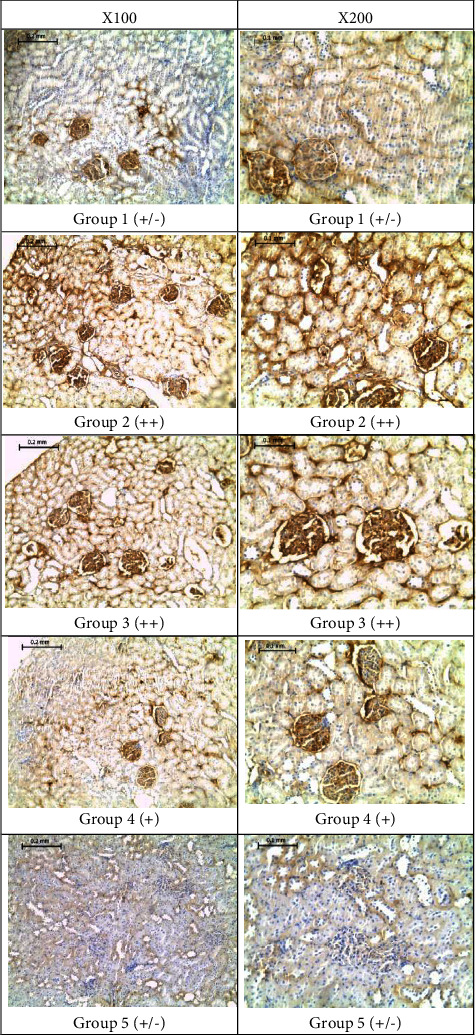
Immunohistochemically stained representative longitudinal sections of the right kidney of rat groups with the corresponding glycation scores at the indicated magnifications.

Figure 4 shows representative images of stained kidney longitudinal sections at two magnifications (100x and 200x). All images were captured for the outer part of the kidney cortex. Kidney sections of the normal rats from group 1 (nondiabetic, vehicle-treated) showed a slightly positive reaction for AGEs (+/−), whereas those of group 2 (diabetic, vehicle-treated) showed a strong positive reaction (++). The VA-treated group 3 (1.5 mg VA/kg) showed a strong positive reaction (++), whereas group 4 (4.5 mg VA/kg) showed a positive reaction (+) and group 5 (15 mg/kg) showed a slightly positive reaction (+/−) that is comparable to that of the nondiabetic group. AGEs were prominently localized in the glomerular endothelial cells, the parietal layer of Bowman's capsules, and the tubular epithelial cells (Supplementary Figure 1). AGEs were most obvious in the diabetic group (group 2) and in group 3, which was treated with the smallest dose of VA (1.5 mg VA/kg). Larger concentrations of VA had remarkably reduced this positive reactivity in kidney tissue in a dose-dependent manner.

### 3.5. VA Reduced AGEs Formation in the Immunohistochemical Assay of the Skin of the Deboned Tails

**Figure 5:**
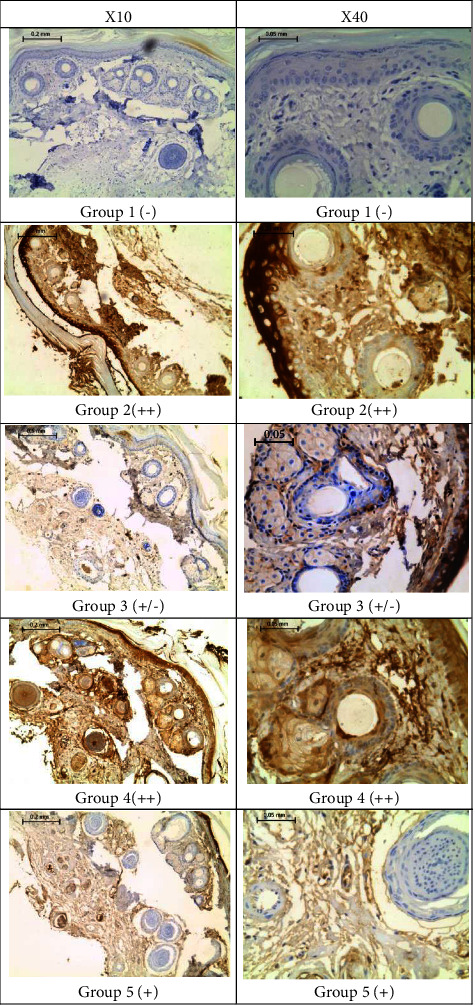
Immunohistochemically stained representative cross-sections of the skin of the deboned tails of rat groups with the corresponding glycation scores at the indicated magnifications.

Figure 5 shows representative images for cross-sections of the skin of the deboned tails. AGEs were prominently localized in the stratum granulosum of the epidermis and the reticular layer of the dermis. Tail sections of control rats (group 1, nondiabetic, vehicle-treated) showed no reaction to the detector antibody (−), whereas those of the diabetic, vehicle-treated rats (group 2) showed a strong positive reaction (++). Unlike the kidney sections, the reactivity of the tail sections of the VA-treated groups varied inconsistently with the VA dose (Supplementary Figure 2). Group 3 (1.5 mg VA/kg) showed a slightly positive reaction (+/−), group 4 (4.5 mg VA/kg) showed a strongly positive reaction (++), and group 5 (15 mg/kg) showed a positive reaction (+).

## 4. Discussion

This study has used in vitro and in vivo experimental models to investigate the antiglycation effect of vanillic acid, a phenolic acid with diverse desirable biological effects [12, 15, 16, 23, 24]. The models used in this study involved glucose-induced BSA glycation, which is widely used in the literature for in vitro glycation, where glycated BSA is considered to be the major product formed [18, 25–27]. The formation of free AGEs as products of glucose binding to constituents other than BSA is unlikely since the experimental milieu consisted of BSA, glucose, and sodium azide dissolved in Na phosphate buffer. In this model, BSA-glucose incubation would generate heterogeneous groups of AGEs, and it would be useful to determine the nonfluorescent AGEs such as CML and MG-H1, which are of clinical significance, but we only focused on the fluorescent AGEs (vs. the nonfluorescent AGEs). Many reports in the literature suggest the use of fluorescent AGEs to detect diabetic and cardiovascular complications. For example, De la Maza et al. suggested using fluorescent AGEs as a screening tool to detect diabetic microvascular complications [28]. Meerwaldt et al. have developed a tool to assess AGE accumulation in diabetic patients by measuring the fluorescence of accumulated AGEs in the skin [29]. Fluorescent AGEs have been used as a marker for assessing the risk of acute coronary syndrome [30], while Yamashita et al. also suggested using fluorescent skin AGEs as a diagnostic marker of neuropsychiatric diseases [31]. In the present study, we used an in vivo model using diabetes-induced glycation in male rats and the cytoprotective effects of VA for RAW 264.7 cells were used to have a better understanding of the vanillic acid potential as antiglycation agent.

In these models, VA has shown notable antiglycation activity and a significant lack of toxicity that can be attributed to a number of possible mechanisms. Glycation reactions involve several species, including electrophilic reactive carbonyl species (RCS) [19], reactive oxygen species (ROS) [32], and transitional metals [33]. These reactions can take place in both the intracellular and extracellular milieu [34].

Interestingly, VA is endowed with chemical features that can combat all of these glycation-inducing factors. These features primarily include acidity, nucleophilicity, lipophilicity, and having a phenolic group. Furthermore, VA has been shown to have many biological activities that can attenuate the level of glycation, which may or may not be mediated by the afore-mentioned chemical features. Among those activities are antioxidative, anti-inflammatory, neuroprotective, and dicarbonyl-trapping activities [12, 16, 24].

Presumably, each of these characteristics of VA plays a pivotal role in its antiglycation actions. Acidity is essential so that VA molecules dissociate at physiological pH, forming negatively charged molecules. The dissociated form of VA, which predominates at physiological pH (the pKa of the carboxylic group of VA is 4.16), results in a negative charge at the oxygen atom in the carboxyl group, which allows complexing with the cationic ions of transitional metals and consequently inhibiting glycation reactions. Lo and his colleagues have also reported that the formed negative charge on phenolic compounds facilitates MGO trapping [35].

Nucleophilicity is essential for scavenging reactive carbonyl species (RCS). VA possesses a hydroxyl group that can provide a nucleophilic center that attacks the electrophilic RCS to form a hemiacetal adduct instead of glycated products [19].

Lipophilicity is also essential from a pharmacological perspective since it allows for the permeation of the compound of interest through the cell membrane to the cytosol, where it majorly exerts its effect. In fact, the intracellular mechanisms of glycation involve various mechanisms that mainly include antioxidation and anti-inflammation, which are mediated by numerous intracellular signaling pathways [34]. Among the reported intracellular events to be affected by VA are the inhibition of Fas-receptor and caspase-mediated apoptosis [15], the reduction of ROS by inducing the endogenous nuclear factor erythroid 2-related factor 2 (Nrf2) and heme oxygenase 1 (HO-1) [16], the inhibition of inflammatory events such as cytokines production, NF*κ*B activation, and expressions of interleukin-1*β* and interleukin-6 [24].

Moreover, the phenol group endows VA and phenolic acids in general with remarkable antioxidative activity since it can stabilize free radicals via a hyperconjugating effect. The electronegative oxygen of the hydroxyl groups of the phenol group enables scavenging free radicals by donating hydrogen atoms to the radicals, thereby stabilizing them [36].

As a phenolic acid, the antiglycation mechanisms reported for phenolic acids can also apply to vanillic acid. It has been shown that several phenolic acids, including ferulic acid, gallic acid, chlorogenic acid, and vanillic acid, can remarkably inhibit glucose-induced protein modification and cross-linking [12]. The antiglycation mechanisms exerted by such phenolic acids mainly include antioxidation [37], anti-inflammation [8], RCS trapping [38], and the intriguing mechanism of protein wrapping [14]. Protein wrapping involves phenol-protein interaction, in which phenols bind to proteins and consequently prevent their interaction with glycating sugars via imposing a steric hindrance effect [14].

Having a myriad of antiglycation properties, VA in this study presumably exerted its antiglycation activity via specific mechanisms that varied according to the conditions of the experimental model used. In the in vitro phase, VA has shown a fair antiglycation activity compared to the positive control AMG (IC50 values of 45.53 mM and 5.09 mM for VA and AMG, respectively, *p* < 0.05). The IC50 value for AMG reported here is about 6 times larger than that reported for antiglycation elsewhere [25]. These authors also reported a much lower IC50 for chlorogenic acid (148.32 *μ*M) using the same BSA-glucose glycation system but for a longer incubation period. This difference in IC50 could be attributed to the lower BSA concentration used by these authors (equivalent to 0.15 mM compared to 0.75 mM in our experiment).

In the present experiments, the toxicity of VA against RAW 264.7 cells was evaluated. VA suppressed MGO toxicity when used in concentrations ranging between 100 and 400 mM (Figure 2). VA is poorly soluble in water; however, it is appreciably soluble in organic solvents. Although VA in the in vitro phase was dissolved in ethanol, which was miscible with the aqueous medium of the reaction, the lipophilicity of VA could enhance its activity in the cell culture system. VA lipophilicity may have facilitated VA penetration through the cell membranes of the cultured cells, where it could exert its effect intracellularly, recruiting multiple antiglycation mechanisms.

In fact, the versatility of the mechanisms through which VA can act in biological (in vivo) versus nonbiological (in vitro) systems should be mindfully considered. In the in vitro system, the antiglycation mechanisms are limited and mainly include RCS-scavenging and antioxidation. On the other hand, the mechanisms involved in the cell system are numerous and much more intricate. In addition to RCS-scavenging and antioxidation, they also include interfering with signal transduction pathways, activating or inhibiting enzymes, chelating metals, blocking AGE receptors, altering cell metabolism, and modulating genetic expression [34].

The lack of toxicity of VA against MGO glycation activity was reported in previous studies using other cell lines and different experimental protocols. Huang et al. [23] found that VA suppressed MGO-induced cell apoptosis in Neuro-2A cells via inhibition of oxidative stress, the MAPK signaling pathway, oxidative-sensitive protein expression, and methylglyoxal-derived carboxymethyllysine formation. This is consistent with our finding that VA significantly reduced MGO-induced cytotoxicity over a wide range of concentrations. Conversely, this finding is nonconcordant with the findings of Lo et al. [35], who reported that phenolic compounds with one hydroxyl group, like VA, have negligible MGO trapping activity. Their work, however, was conducted using a cell-free experimental system.

For the in vivo system, VA showed no inhibitory effect on blood fructosamine or HbA1c. Fructosamine, as well as HbA1c, tests are clinically approved to monitor diabetes in diabetic patients [39], and we also substantiated these tests with the immunohistochemical study (IHC assay). Although fructosamine is an Amadori product and may be thought of as not stable, the Amadori reaction comes after the Schiff base reaction and produces more stable products [40]. Moreover, the rate of reversibility of the Amadori reaction is practically insignificant, and the products of the reaction are considered stable [41].

In our work, VA caused a less remarkable effect on blood fructosamine and HbA1c compared to the kidney and tail tissues (Figures 4–5). This variation in VA activity can be attributed, at least in part, to the variation in the distribution of VA among different tissues. This presumption is supported by a study that investigated the tissue distribution of VA and other bioactive constituents of two Chinese herbs in rats, where VA was found to be mostly distributed in the liver and kidney [42]. Being a lipophilic compound, this variation in tissue distribution is expected and may explain the greater exhibition of VA activity in kidney tissues compared to blood proteins in the current study.

In an observable note, VA did not exhibit a hypoglycemic effect in the treated diabetic rats in this study (data not shown). Thus, serum, where fructosamine is formed, and RBCs, where hemoglobin is found, were not protected from exposure to the high blood glucose level experienced by the diabetic rats. RBCs are expected to be subjected to more intensive flux of glucose compared to kidneys, since the abundant glucose transporter in the RBCs, GLUT-1, has a higher affinity to glucose compared to the abundant glucose transporters in kidney, SGLT-1 and GLUT-2 [43].

Another possible explanation of this discrepancy in VA activity between blood and kidney tissues can be attributed to the mediatory mechanisms operating in each tissue. Fructosamine represents glucose-bound serum proteins, mainly albumin and other serum globulins [44]. Fructosamine is a noncellular compound, and thus the mechanisms preventing its formation involve only limited noncellular mechanisms such as antioxidation, nucleophilic attack, and metal chelation. Regarding HbA1c, although hemoglobin is entrapped inside RBCs, mature RBCs can be viewed as sacs with hemoglobin filling the majority of their volume. In fact, the mechanism of hemoglobin glycation is very well established as a glycosylation reaction that involves spontaneous reaction with circulating blood glucose without much implication of RBCs [45]. Consequently, the intracellular mechanisms of antiglycation that may take place in typical body cells are not expected to act in atypical cells like RBCs. On the other hand, for the kidney tissues, the mechanisms that involve intracellular events such as anti-inflammation, signal transduction modification, enzymatic activation or inhibition, receptor blocking, gene modulation, and others may have taken place, outweighing the impact of VA in serum and RBCs.

Compared to kidney tissues, albumin and RBCs are also known for their relatively rapid regeneration and short-lived proteins. This may have restrained the full exertion of VA antiglycation activities, particularly the late-acting ones. As expected, long-lived proteins give the chance for the late antiglycation mechanisms, which proceed in the advanced stages of glycation, to come into action sometimes under nonpathological conditions, albeit at a substantially slower rate [46]. Kidney and skin tissues are rich in long-lived proteins such as collagen and keratin, a matter that may have allowed for the presumed late-acting antiglycation mechanisms of VA to take place and intensify its observed effect there. Collagen, for example, is one of the longest-lived body proteins, with an average half-life extending for years; thus, it is highly susceptible to extensive and advanced glycation [47].

The localization of AGEs in the glomerular endothelium in the present study is consistent with the findings of Ling et al. [48] and Scott et al. [49], who detected an intensely positive reactivity in the glomerular epithelium of diabetic rats to various AGEs-specific antibodies. In support of the antiglycation effect of VA on the kidney, a recent study investigating the nephroprotective effect of vanillic acid against cisplatin-induced nephrotoxicity in Wister rats found a remarkable nephroprotective effect of vanillic acid [50]. The researchers have attributed this effect to the antioxidative and anti-inflammatory potential of VA.

In contrast to our findings, other researchers have found that phenolic acids like ellagic acid inhibit the formation of glycosylated Hb in humans significantly. This difference between the activities of ellagic acid and VA can be attributed to the fact that ellagic acid is a poly-hydroxylated acid and thus has a more potent antioxidative capacity compared to the monohydroxylated VA [35, 51].

Interestingly, VA was found to have no toxic effect on the treated RAW 264.7 cells (Figure 2). At the in vivo level, VA has also enjoyed a high tolerable level. The LD50 of VA for rats was found to be 5000 mg/kg [52], a dose that is several hundred folds higher than the doses used in our study, which ranged from 1.5 to 15 mg/kg. This observation supports the use of VA for therapeutic purposes.

Unlike kidney sections, the images of the skin of the deboned tail sections did not show a consistent gradient in the intensity of the stain representing AGEs. Nevertheless, an obvious regression in stain intensity is recognized in the sections treated with the highest VA dose, a matter that is consistent with staining patterns in the sections of the kidney. A recent review has found that the intervention period needed to observe changes in AGEs levels in the skin, bones, and muscles of experimental rats varied from 10 to 32 weeks [6], suggesting that a longer intervention period should have been practiced in order to observe more consistent changes in AGEs levels in the skin of the deboned tails of the rats in this study.

## 5. Conclusion

The results of this study demonstrated that vanillic acid has considerable antiglycation activity, particularly at the level of cellular, nonblood, long-term glycation. Along with the reported beneficial effects of VA as a potent antioxidant and anti-inflammatory agent, aside from the notable biological safety of the compound, the use of VA as a promising antiglycation candidate should be considered for further pharmacological investigation and medical use. Further studies that investigate the potential of VA and other phenolic acids as adjunctive therapies for glycation-related maladies are highly warranted.

## Data Availability

All the data used in this work are embedded in the manuscript and the supplementary figures appended to it and are available from Dr. Al-Hadid or from the corresponding author upon request.

## Supplementary Materials

Supplementary Materials Supplementary Figure 1: immunohistochemical detection and localization of AGEs in longitudinal sections of the kidneys of the experimental rats. (a) Cross-section of arteriosclerotic plaque of a 72-year-old patient, which was used as a positive control. (b)–(f) Sections of the five animal groups, and they were all taken for the outer part of the kidney cortex. The intensity of the brown color reflects the level of AGEs in the tissue and is symbolized by the designated score in each image (see text for detailed grading of tissues). The antibody used for the assay was polyclonal AGE-antibody (ab23722, Abcam, USA). VA, vanillic acid; BC, Bowman's capsule; PCT, proximal convoluted tubule; G, glomerulus. The tips of the solid arrows point at the parietal layer of BC, whereas those of the dashed arrows point at the tubular epithelial cells. Original magnification (200x). Supplementary Figure 2: immunohistochemical detection and localization of AGEs in cross-sections of deboned tails of the experimental rats. (a) Cross-section of arteriosclerotic plaque of a 72-year-old patient, which was used as a positive control. (b)–(f) Sections of the five animal groups. The intensity of the brown color reflects the level of AGEs in the tissue and is symbolized by the designated score in each image (see text for detailed grading of tissues). The antibody used for the assay was polyclonal AGE-antibody (ab23722, Abcam, USA). VA, vanillic acid; SG, stratum granulosum; HF, hair follicle; RR, reticular region. Original magnification (100x).
