# Supplementary figures and images for: Vanillic Acid Inhibited the Induced Glycation Using In Vitro and In Vivo Models
Source: Evid Based Complement Alternat Med. 2022 Nov 18;2022:7119256. doi: 10.1155/2022/7119256 (PMC9699731; doi:10.1155/2022/7119256)

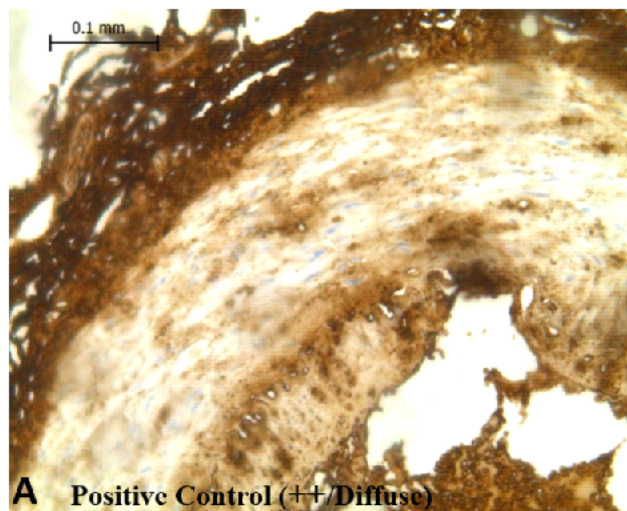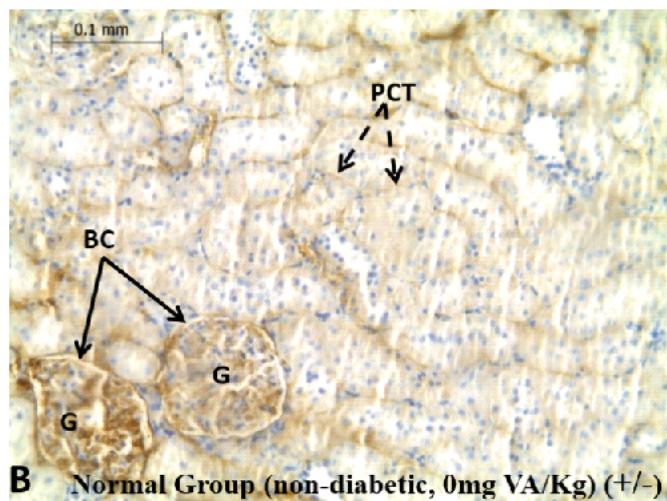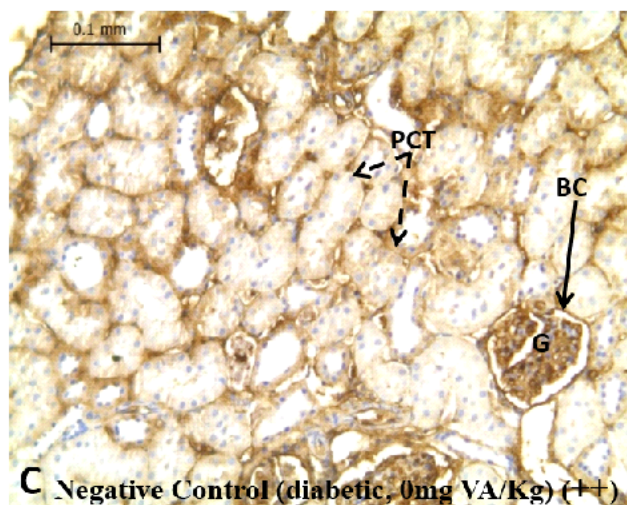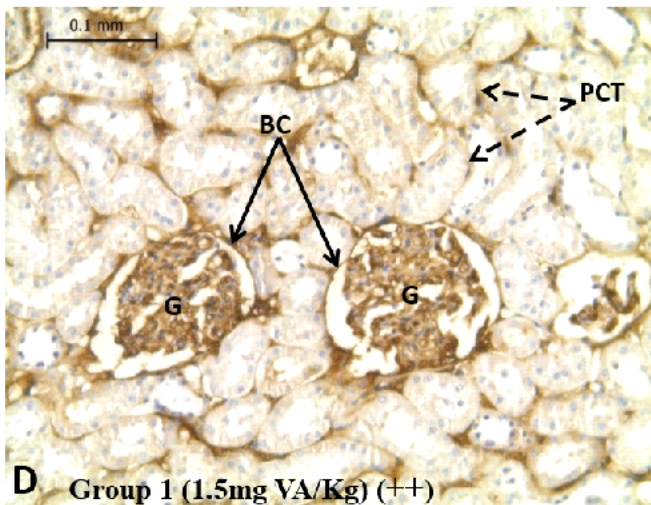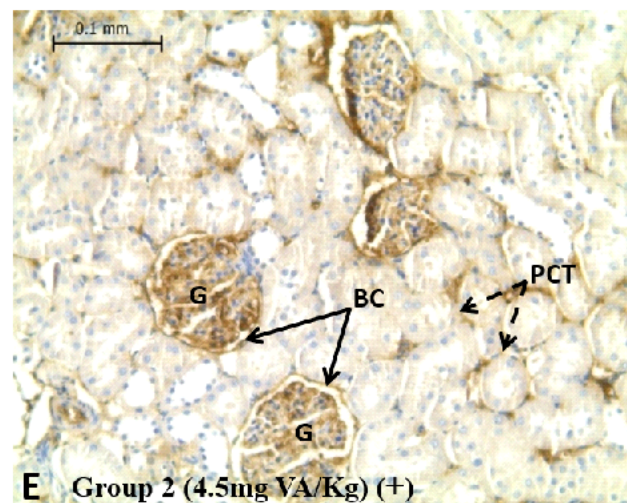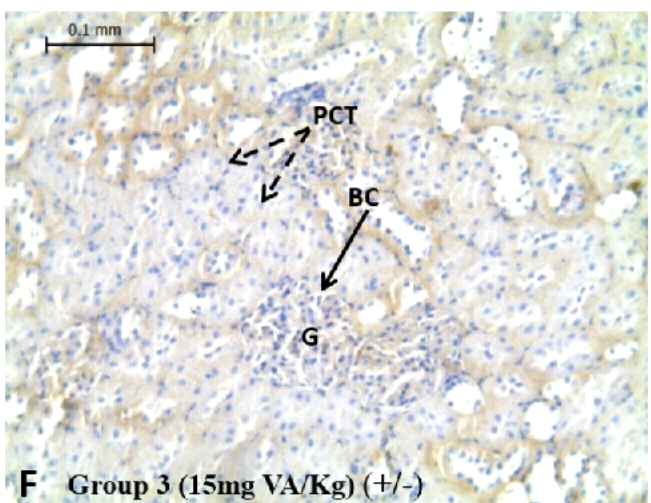

Supplement: Supplementary Materials — Supplementary Figure 1: immunohistochemical detection and localization of AGEs in longitudinal sections of the kidneys of the experimental rats. (a) Cross-section of arteriosclerotic plaque of a 72-year-old patient, which was used as a positive control. (b)–(f) Sections of the five animal groups, and they were all taken for the outer part of the kidney cortex. The intensity of the brown color reflects the level of AGEs in the tissue and is symbolized by the designated score in each image (see text for detailed grading of tissues). The antibody used for the assay was polyclonal AGE-antibody (ab23722, Abcam, USA). VA, vanillic acid; BC, Bowman's capsule; PCT, proximal convoluted tubule; G, glomerulus. The tips of the solid arrows point at the parietal layer of BC, whereas those of the dashed arrows point at the tubular epithelial cells. Original magnification (200x). Supplementary Figure 2: immunohistochemical detection and localization of AGEs in cross-sections of deboned tails of the experimental rats. (a) Cross-section of arteriosclerotic plaque of a 72-year-old patient, which was used as a positive control. (b)–(f) Sections of the five animal groups. The intensity of the brown color reflects the level of AGEs in the tissue and is symbolized by the designated score in each image (see text for detailed grading of tissues). The antibody used for the assay was polyclonal AGE-antibody (ab23722, Abcam, USA). VA, vanillic acid; SG, stratum granulosum; HF, hair follicle; RR, reticular region. Original magnification (100x). [file 7119256.f1.zip › Figure-1-supplemantry-.pdf]
